# Yeast as a By-Product from Wine and Beer Production: Comparative Evaluation of Physico-Chemical Composition

**DOI:** 10.3390/molecules31020280

**Published:** 2026-01-13

**Authors:** Ionuț Avrămia, Adriana Dabija, Mircea Oroian, Larisa Caisin, Vitalii Agapii, Aurelian Rotaru, Ancuta Chetrariu

**Affiliations:** 1 Faculty of Food Engineering, Stefan cel Mare University of Suceava, 720229 Suceava, Romania; adriana.dabija@fia.usv.ro (A.D.); m.oroian@fia.usv.ro (M.O.); ancuta.chetrariu@fia.usv.ro (A.C.); 2 Department of Animal Science and Biotechnology, The State Agrarian University of Moldova, bd. Ștefan cel Mare, 168, MD-2004 Chisinau, Moldova; larisa.caisin@mpasa.utm.md (L.C.); vitalii.agapii@gmail.com (V.A.); 3 Faculty of Electrical Engineering and Computer Science & Research Center MANSiD, Stefan cel Mare University of Suceava, University Street, No. 13, 720229 Suceava, Romania; aurelian.rotaru@usm.ro

**Keywords:** spent brewer’s yeast, wine lees, freeze-drying, oven-drying, circular economy

## Abstract

The trend toward sustainable protein substitutes is driven by growing concerns about food security, sustainability, and human health. Spent brewer’s yeast and wine lees are two by-products of the beer and wine industry with high potential, owing to their complex composition, which remains insufficiently exploited. The purpose of this study was to perform a comparative analysis of the two by-products under two different drying techniques to observe if there are significant changes in composition: oven-drying and freeze-drying. Two samples of wine lees from producers in the Republic of Moldova were used—Asconi Winery and Cricova Winery (Republic of Moldova)—as well as a sample of spent brewer’s yeast offered by Efes Vitanta Moldova Brewery. The samples were characterized in terms of physicochemical properties, antioxidant activity (total polyphenol content (TPC), individual polyphenol content, and DPPH assay scavenging activity), color, mineral content, structural composition (FT-IR analysis), and microstructure, as well as organic acid and B vitamin content. The highest protein content was recorded in the samples from Cricova (45.35–46.81%). Regarding the polyphenols, the oven-dried Efes sample exhibited a TPC value of 3.98 mg GAE/g, while the highest DPPH value of 88.92% was observed in the Asconi sample. All analyzed samples showed a diverse composition of individual phenolic compounds, including 4-hydroxybenzoic acid, vanillic acid, caffeic acid, and rosmarinic acid. Wine lees samples have the highest content of B vitamins, with vitamin B3 being the most abundant across all samples, followed by vitamin B6. The microstructural examination revealed autolyzed yeast cells, with more permeable cell walls, favorable to subsequent valorization treatments, and in some cases, cells form clusters in a mother-daughter junction due to serial re-pitching.

## 1. Introduction

By 2050, global resource consumption is expected to reach three times the available supply. To counteract this trend, the European Union aims to implement strategies such as the circular economy, which focuses on reducing and recycling by-products. These challenges are particularly evident in the agro-industrial industry, one of the main generators of waste worldwide. When mismanaged, agro-industrial residues can lead to serious environmental issues, including greenhouse emissions and oxygen depletion [1]. Within this context, waste yeasts, a by-product of the fermentation industry, represent a promising resource. Recent studies highlight that waste yeasts possess valuable nutritional and physicochemical properties, suggesting opportunities for their use in diverse applications, such as functional food ingredients [2,3,4].

Spent brewer’s yeast (SBY) and wine lees (WL) are major by-products of the brewing and winemaking industries, generating hundreds of thousands of tons of biomass with high nutritional and economic potential annually. These by-products contain significant amounts of proteins, carbohydrates, B vitamins, and valuable bioactive compounds (e.g., β-glucans, mannoproteins, polyphenols). Their recovery contributes not only to reducing the volume of organic waste, but also to promoting the circular bioeconomy, having a positive impact on the environment by reducing greenhouse gas emissions, chemical oxygen demand, and biological oxygen demand [5,6,7,8].

Globally, the brewing industry produces approximately 1.86 billion hL of beer annually, generating an average of 1.5–3 kg of SBY/100 L of beer, i.e., a total estimated between 279,000 tons and 558,000 tons of residual yeast. Traditionally, brewer’s yeast is used in serial repitching up to six times to reduce costs, or in animal nutrition due to its protein profile. However, new reuses of this by-product are being sought, such as using it as a fertilizer or a material for obtaining biogas, or in the food industry as a functional ingredient [3]. In the wine industry, wine lees represent 2–6% of the total wine volume produced, representing the second most important by-product, with an estimated contribution of approximately 25% of total waste. Wine production generates about 60 kg of residual yeast/tonnes of grapes, leading to a global estimate of 2 million tons of residual yeast/year, while in brewing, yeast residues represent up to 15% of the total resulting by-products [4,9,10,11].

In terms of chemical composition, SBY contains approximately 45–60% (in some cases up to 70%) proteins, 25–59% carbohydrates, and 0.7–1.5% lipids, mainly palmitic and oleic fatty acids. It is also a source of minerals (phosphorus, potassium, sodium, calcium, magnesium, and zinc) and B-complex vitamins (B7, B3, B6, and B5). The carbohydrates are composed primarily of β-glucans (50–60%), mannoproteins (35–40%), chitin (1–3%), and glycogen (1–23%). SBY also has a high energy value with nutraceutical and functional potential [4,9,12]. Its chemical profile is influenced by multiple factors, including the yeast strain used as a starter, the general fermentation conditions and brewing process, the number of yeast reuses, and the yeast separation process [5].

The chemical composition of WL includes 20–30% proteins, containing essential amino acids such as tyrosine and valine; 30–50% carbohydrates, predominantly insoluble fiber, cellulose, and hemicellulose; and 5–10% lipids, rich in valuable fatty acids such as palmitic, linoleic, and stearic acids. In addition, WL contains 26–245 mg GAE/g polyphenols, mainly flavonoids, stilbenes, and anthocyanins, as well as organic acids: tartaric acid, gallic acid, ellagic acid, or mineral substances (K, Ca, Mg, Fe, and Zn) [8,11,13,14,15]. According to data from the literature, WL shows composition heterogeneity, as many parameters depend on various factors such as winemaking method, types of yeast and grapes used, or the characteristics of the cultivation soil [13].

To date, several approaches for the valorization of the two types of residual yeasts have been reported in the specialized literature. SBY has been used in the food industry as a functional ingredient (stabilizer, texturizing agent, and flavor enhancer), and in the form of yeast extracts in various food products—ham, mayonnaise, yogurts, and soups—while the extracted β-glucans and mannoproteins can have different applications, being approved as generally considered safe for human consumption (GRAS) by the FDA [3,5,16]. However, the high level of RNA nucleotide bases, including purines, present in the raw material is a limiting factor that prevents the use of a significant proportion of SBY in food products. Increased uric acid in the bloodstream can be caused by excessive purine consumption, which has harmful effects on human health. Although this remains a significant obstacle, purines can be removed through additional processes such as thermal, enzymatic, or alkaline hydrolysis treatments. Further studies are therefore needed to optimize the use of these technologies in SBY processing [5,17].

A classical valorization pathway identified for SBY is its use in animal nutrition, where it provides significant functional and nutritional benefits. SBY has been used as a protein supplement for ruminants, contributing to increased milk production and improved digestion. Under in vitro conditions, SBY, rich in antimicrobial α- and β-acids, reduces excessive degradation of proteins in the rumen by inhibiting hyperammoniacogenic bacteria. In the case of sheep, complete replacement of corn meal with SBY (100%) produced no negative effects on consumption, feeding behavior, or digestibility [2]. In broilers, replacing the vitamin premix with sun-dried SBY led to significant weight gain and higher live weight, especially in Indian River breeds. Up to 75% fishmeal substitution has been experimented with in aquaculture [3,16].

Other applications of SBY have been explored in the cosmetic and pharmaceutical industries, where it serves as a valuable source of antioxidants, peptides, vitamins, and bioactive polysaccharides for use in dermocosmetic products and supplements. Additionally, SBY has been used in the production of bioethanol, succinic acid, and 2-phenylethanol, as well as a biosorbent for wastewater treatment and a biomass source for insects, among other uses [3,9].

Various bioactive compounds have been extracted from wine lees, including polyphenols (with antioxidant, antimicrobial, and anti-inflammatory properties), anthocyanins (malvidin and peonidin), stilbenes (resveratrol), tartaric acid, ethanol, gallic acid, β-glucans, and mannoproteins. In the food industry, these compounds have also been used for the fortification of cereal bars, ice cream, and meat preparations as an antioxidant and natural preservative or as a source of nitrogen and vitamins. Other applications of WL include composting, biostimulants, and anaerobic digestion for biogas production, and as a support for microalgae producing biofuels. In the pharmaceutical and cosmetic industries, phenolic extracts showed antineoplastic, anti-inflammatory, and photoprotective activity, being applied in dermatocosmetic formulations with antineoplastic and skin-protective properties [8,18,19,20]. In nutrition, supplementing with wine by-products rich in polyphenols and vitamin E has been shown to improve oxidative balance both at the systemic level and in the quality of meat products. Although WL itself is relatively low in antioxidants, it has demonstrated antioxidant effects, possibly through supporting intestinal integrity. However, the absorption mechanisms of polyphenols are complex, which can negatively affect their bioaccessibility and bioavailability, and thus requires further research. While polyphenols possess significant antioxidant potential, their practical applicability in animal nutrition, as an alternative to synthetic additives and growth promoters, remains insufficiently explored, despite the existence of similar commercial products [21].

The valorization of spent yeast is essential in the context of the circular bioeconomy. SBY presents a high potential as a source of protein and nutraceutical with multiple applications. However, its direct use in human nutrition is limited by its high nucleic acid content. WL is rich in valuable bioactive compounds but is currently poorly exploited industrially. Modern extraction technologies combine processing efficiency with sustainable development principles [8,9]. Neverless, the efficient industrial integration of both types of yeast requires the development of robust, scalable, and environmentally friendly technologies [22].

Promoting new value chains based on waste yeast can contribute to reducing disposal costs, developing innovative products, and promoting sustainable growth of the agri-food and biotech sectors. The full valorisation of these resources is a strategic direction for achieving the objectives of the European Green Deal and the UN 2030 Agenda for Sustainable Development.

The paper presents a comparative study of the physicochemical composition of the two types of residual yeasts, SBY and WL, aiming to identify optimal solutions for valorizing the valuable compounds they contain. A study on drying using freeze-drying and the spray-drying effect of mannans from *S. cerevisiae* did not identify significant variations between the two drying types except for color parameters, and differences in moisture content and solubility, which determine the drying method to be used based on the final use of the material [23]. The two types of drying have several advantages and disadvantages, the most important of which are the preservation of the structure and bioactive properties; the chemical components of the dried material undergo minimal oxidative and chemical degradation, and due to the low moisture, the storage time is prolonged in the case of freeze-drying. On the other hand, drying in an oven comes with the advantage of a shorter drying time. With the correct equipment, the main disadvantages are high costs in the case of freeze-drying, and the need for heat-stable materials in the case of oven-drying. More studies are needed to analyze the two types of drying and their advantages for some samples, and also how certain aspects can be improved.

## 2. Results and Discussions

### 2.1. Proximate Analysis, Total Phenolic Content (TPC), and DPPH Assay Scavenging Activity

The results of proximate analysis, total phenolic content, and antioxidant activity are presented in Table 1.

Table 1 shows a variable ash content in the yeast samples dried by the two methods, freeze-drying and oven-drying, ranging from 3.86 to 6.69%. In spent brewer’s yeast, in addition to its natural composition, ash between 5.64 and 6.69% can occur from the production water, from mineral impurities, or from the cereals used. In contrast, in wine lees, mineral residues come mainly from clarifying agents, soil mineral uptake, processing additives used during winemaking, or from the fiber content. These results are consistent with those reported by Caballero-Cordoba & Sgarbieri (2000) and Marson et al. (2020) [12,24]. Previous studies have reported a high content of up to 10.39% in autolyzed yeast samples from SBY [25]. Avrămia & Amariei (2021) and Caponio et al. (2024) reported that yeast residues from brewing contain approximately 1% lipid, which aligns with the results obtained in this study [9,26].

The protein content of yeast varies widely, ranging from 12.86 to 46.81%, with a low content in the Asconi samples, where the fiber content is slightly high. Jach et al. (2022) highlighted the protein from various types of yeast as an alternative nutritional source [27]. In this context, the different yeast species from specific residual substrates presented a protein content ranging between 26% (in *Candida utilis* isolated from salad oil wastewater) and 70.4% (in *Candida* sp. isolated from prawn-shell waste). For *S. cerevisiae*, the content ranged between 33 and 54%, with 49% in the isolates from the brewing industry [27], a percentage that aligns with the results from SBY and C-OD and C-FD. The protein content of these samples is relevant for selecting applications in preparing protein concentrates with a functional role, as described by Haldar et al. (2011), who reported superior production performance in heat-stressed broilers [28]. Furthermore, Cao et al. (2025) compared yeast protein with proteins from plant sources (soybean, wheat, and pea) and animal sources (whey protein) in rat-feeding trials, concluding that yeast protein possesses an adequate amino acid composition, moderate digestibility, and the capacity to balance amino acids in plant-based foods [29].

The total polyphenol content (TPC) was highest in the spent brewer’s yeast samples, followed by the wine lees samples. The TPC ranged from 1.57 to 3.98 mg GAE/g with the highest content observed in the spent brewer’s yeast samples. Previous reports have indicated contents of approximately 0.21 mg GAE/g in spent brewer’s yeast using HPLC methods [30] and between 3.1 and 40.9 mg GAE/g in different types of wine lees, with the Pinot Noir freeze-dried lees at the highest pole [31]. The drying method does not significantly influence these results, which are in agreement with the data reported by Caponio et al. (2024) [26] and Gagliano et al. (2025) [10]. On the other hand, De Luca et al. (2023) reported increased TPC values, influenced by the solvent and the extraction method [32]. Cotârleț et al. (2025) analyzed freeze-dried wine lees subjected to several extraction methods, obtaining results in the range of 0.98 ± 0.005–2.90 ± 0.13 mg GAE/g dry matter, values close to those obtained in this study [11]. All these characteristics may be due to the type of wine lees, which depends on the variety and origin of the grapes, the winemaking process, and the extraction methodology. The samples exhibited similar DPPH scavenging activity values, which were not significantly affected by the drying method. These results align with those reported by Caponio et al., 2024 [26]. The DPPH results indicate stronger antioxidant activity for A-FD, A-OD, and C-FD samples, while the oven-dried samples of Cricova and Efes, and the freeze-dried spent brewer’s yeast, showed values below 50%. A slightly increased percentage of 59.7% in spent brewer’s yeast extract was found by [30]. Despite a low TPC, the high DPPH radical scavenging activity confirms the presence of other non-phenolic antioxidant compounds (e.g., peptides, polysaccharides derived from the yeast cell wall, Maillard reaction products, or melanoidins).

### 2.2. Individual Phenolic Compound

Wine and wine by-products are rich sources of phenolic compounds, including coumaric, ferulic, caffeic, gallic, and rosmarinic acid. The Cricova and Asconi samples exhibited higher values for these samples when processed by the freeze-drying method, as shown in Table 2.

WL showed a higher content of individual phenolic compounds; notably, 4-hydroxybenzoic acid, rosmarinic acid, vanillic acid, p-coumaric acid, and caffeic acid stand out, in agreement with the data reported by Jurcevic et al. (2017) [33]. Low amounts of quercetin and kaempferol were also reported in the WL of the Asconi samples. In contrast, SBY contains phenolic compounds derived primarily from barley and hops, but at lower levels compared to WL. Some interactions between phenolic compounds and other constituents (proteins and polysaccharides) can lead to their partial precipitation, which also reduces the Folin–Ciocalteu reaction [34]. The individual phenol content is influenced by several parameters, including the raw materials used in the wine and beer production process, the fermentation conditions, or the drying conditions of the by-products.

### 2.3. FT-IR Analysis

**Figure 1:**
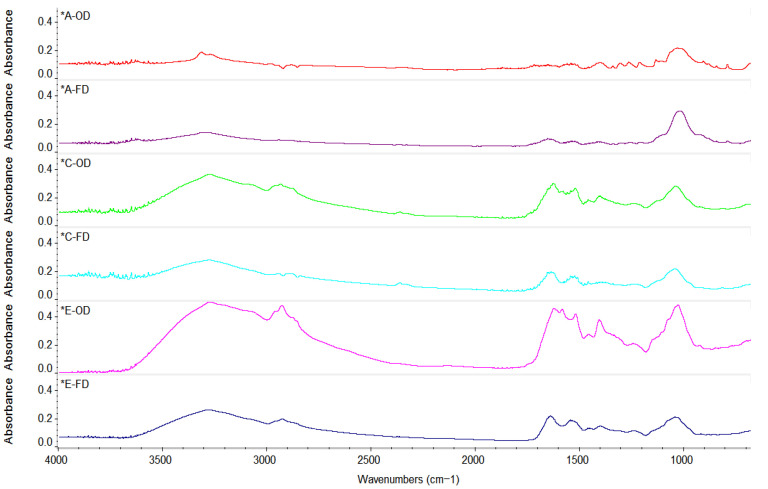
FT-IR spectra of the yeast samples. The asterisk preceding the sample code name indicates the ATR correction.

The FT-IR spectra of WL and SBY samples recorded in the range of 650–4000 cm^−1^ are presented in Figure 1. The spectra show absorption bands around 3300 cm^−1^, 1600 cm^−1^, and 1000 cm^−1^, specific to O-H stretching and the carboxylate and carboxyl functional groups, respectively [35]. De Luca et al. (2023), in a comparative analysis of different extraction treatments applied to wine lees (water, alcohol or hydroalcoholic extractions), reported characteristic absorption bands of phenolic compounds in the range 1850–450 cm^−1^. Specifically, up to 1200 cm^−1^ and from 1400 to 1650 cm^−1^, the authors identified, in the fingerprint region, benzo-γ-pyrone and benzopyrylium signals associated with flavonoid fragments. Furthermore, carboxyl group vibrations were observed near 1755 cm^−1^, while methoxyl groups characteristic of flavonoids were detected between 950 and 1470 cm^−1^ [32]. A hypsochromic shift to the O-H stretching vibration was observed in the Cricova and Efes freeze-dried samples compared to the oven-dried ones. Additionally, Efes samples exhibited higher absorbance in the 1400–1700 cm^−1^ range, indicating a more intense vibrational transition.

### 2.4. Determination of Complex B Vitamins Content

It is well known from the literature that yeast can retain a portion of the vitamins present in the environment in which it develops. Methner et al. (2022), to verify previous data and clarify whether yeast absorbs vitamins or/and immediately metabolizes them from the wort, reported double values of thiamine concentration in brewer’s yeast sediment (33 µg/g). From an initial 330 µg/L thiamine content in the wort, the yeast absorbed approximately 240 ug/L [36].

In this work, we conducted research on the simultaneous separation of six vitamers (thiamine hydrochloride, riboflavin, nicotinamide, pyridoxine hydrochloride, biotin, and cyanocobalamin) from both spent brewer’s yeast and wine lees, following previously established protocols.

**Figure 2:**
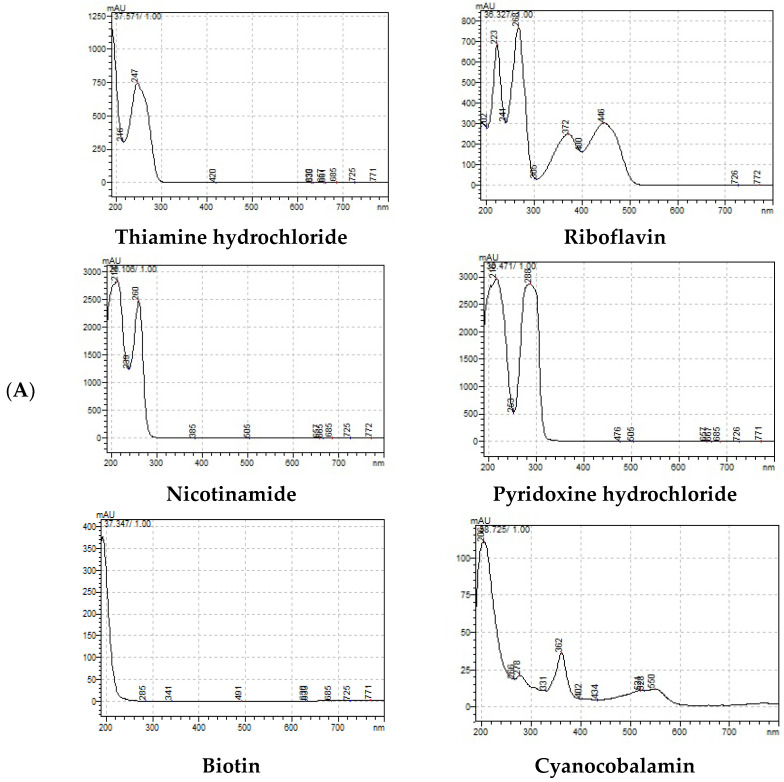

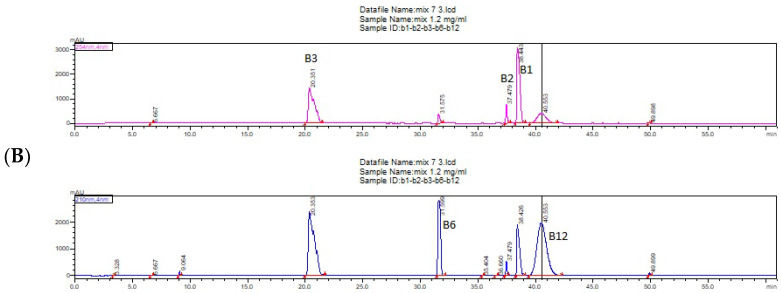
HPLC chromatogram of working standards: (**A**) UV-vis absorbance and retention time for the six individual vitamins (B1, B2, B3, B6, B7, and B12); (**B**) Standard mixture solution in the two wavelengths (210 and 254 nm).

Four HPLC preliminary work protocols were used in this study with potassium acetate, phosphate buffer, methanol, or ammonium acetate as mobile phases. From the above tests, it was concluded that these mobile phases could not separate B vitamins in a single run. Among those described by [37,38,39,40], the only one that led to conclusive results was that of Vidović et al. (2008), where the mobile phase containing hexane 1-sulfonic acid sodium salt became attractive in the chromatographic separation and helped in the formation of ion pairs, increased the retention on the column, and otherwise improved the separation between similar vitamins [38]. The elution was observed at 254 nm for B1, B2, and B3, and at 210 nm for B6, B7, and B12 (Figure 2). Vitamer identification was performed based on the relative order of the peaks, confirmed with standards analyzed under the same working conditions.

The results obtained are presented in Table 3, where 5 out of 6 water-soluble vitamins evaluated could be quantified, except for B12, which was below the detection limit (80 µg/mL) and generated a low signal-to-noise ratio (S/N) leading to inaccurate detection and quantification (signal obscured by baseline noise). Mateeva et al. (2023) identified traces of this compound, and their statements were limited to the fact that a relatively higher content of vitamin B12 would have required additional processing of the samples [39]. Low values of cyanocobalamin content were also tabulated by Puligundla et al. (2020) of <0.25 mg/100 g, and by Czech et al. (2016) of 60 µg/kg in *Yarrowia lipolytica*, suggesting that yeast does not naturally contain cyanocobalamin [16,41]. Indeed, such comparative studies, focused on a single vitamin extraction, were conducted by Rocchi et al. (2022) on thiamine from fresh yeast biomass. The authors concluded that thiamine pyrophosphate degradation can be minimized by bead beating and moderate hot hydrolysis, avoiding exposure to extreme temperatures. The highest vitamin B1 content identified after optimal treatment was 71.8 nmol/g DW [42], equivalent to approximately 0.0238 mg/g DW thiamine. The present study identified vitamin B1 in concentrations between 0.0274 and 0.0904 mg/g, with the highest values in freeze-dried samples compared to oven-dried ones.

Riboflavin, on the other hand, varies in fairly wide lines between 0.0196 and 0.0795 mg/g, also with increased or slightly increased values in lyophilized samples. 0.0112–0.0355 mg/g B2 was identified by Demirgul et al. (2022) in 3 different yeast species from sourdough, with the highest content in *S. cerevisiae*, while Jaeger et al. (2020), in a review on the possibilities of valorization of spent brewer’s yeast, presented three sets of data on vitamin B content from different authors, with the highest content in B2 of 10.6 mg/100 g DW (0.1060 mg/g B2) [2,43]. Varga and Maraz (2002), in a study on the cellular distribution of Fe, Zn, Se and Cr in yeast cells, suggested that iron enrichment of yeast cells leads to an increase of up to 3-times higher B2 (if we also analyze the data provided by Czech et al. (2016) from Table 3 by comparing the iron content and B2, the conclusions will be the same) [41,44]. However, over the storage period (12 months), this content decreased to 13 µg/g (62%) and to 14 µg/g for B1 [44]. Our results also showed an increased trend of riboflavin in samples that showed a higher iron content (A-OD, E-OD, and E-FD). It is also worth mentioning the research of Hälvin et al. (2013) who, by applying four different extraction treatments, identified the highest values of thiamine and riboflavin by extraction with an enzymatic mix of 104 µg/g B1 compared to 34 µg/g without any treatment, and 75 µg/g compared to 12 µg/g [45]. Vitamin B3 showed important variations (0.8667–2.3845 mg/g) and was found in the highest amount in wine lees, A-OD, and A-FD samples. A similarly high vitamin B3 content of 1.2 mg/g was also discovered by Mateeva et al. (2023) in brewer’s yeast, while other studies such as Vieira et al. (2016) obtained values of 77.2 mg/100 g DW [30,39]. Regarding vitamin B6, Minami et al. (1982) stated that vitamin B6 content is affected by the presence of thiamine [46], although the present study did not find a correlation between these two sets of data. Vitamin B6 ranged between 0.0499 mg/g and 0.1230 mg/g with increased values in A-FD and E-OD samples. Biotin, on the other hand, showed losses after the freeze-drying process in all three types of yeast, with values ranging between 0.0095 and 0.0296 mg/g. Biotin is quite stable during freezing or frozen storage; however, we could not find a scientific explanation in the current literature for that loss during the freeze-drying process. Most likely, the oscillating temperatures led to this loss. Other studies have reported values of up to 454.11 mg/100 g biotin in yeast isolated from sourdough [43]. The variations in compositional changes found, alongside the predominant preservation of vitamins in lyophilized samples, are well-documented in the scientific literature. Thus, a study on the impact of lyophilization on the bioactivity and physical properties of food products in 2024 identified that lyophilization also preserves the flavor of the samples, in addition to preserving the original structure of the bioactive compounds (as well as for vitamin C) [47]. A study on kale leaves performed by Korus Anna (2021), comparing air-drying and freeze-drying, concluded that freeze-dried kale retained much higher levels of B-vitamins than air-dried kale [48]. Moreover, in the same study, after 12 months of storage, the content of vitamin B1 and B2 in the freeze-dried samples was modest compared to the air-dried samples (1–4% of B2 in FD samples compared to 8–16% OD). Also, HPLC verification of the stability of vitamins in freeze-dried fortified military foods for a longer period of time, 24 months, concluded that vitamin B1 is maintained up to 94%, B2 at 97%, B6 at 86%, and vitamin E at 77%, finding freeze-drying reliable and with a high degree of protection for food [49]. Consequently, the evidence indicates that a significant influence is present regarding freeze-drying and oven-drying, due to the distinct way in which the two techniques remove water and the associated thermal conditions, which determine different effects on the composition and microstructure of the material. Compared to the conventional oven-drying method, freeze-drying is one of the most advanced drying methods, which removes water from the frozen material mainly by sublimation. High temperatures inherent to conventional drying reduce product quality, whereas the lower processing temperatures used in freeze-drying enhance both quality and stability by minimizing the degradation of heat-labile constituents, including vitamins, antioxidants, and aromatic compounds.

### 2.5. Color

**Figure 3:**
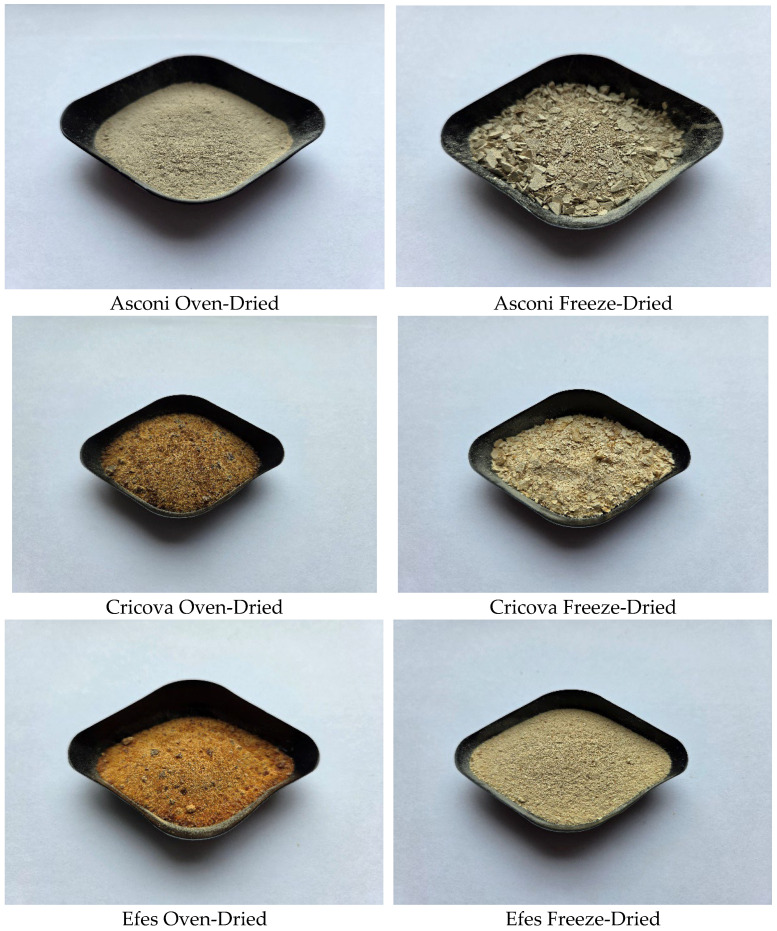
Digital images of lees and spent brewer’s yeast samples dried by the two methods.

Figure 3 shows the samples dried by two methods: oven-drying and freeze-drying. Visual differences can be observed between the two drying methods; however, the drying method used is not the only thing that affects the color of the samples.

Color parameters L (lightness), a* (redness), and b* (yellowness) were evaluated in three different points after using a Konica Minolta CR-400 (Tokyo, Japan) portable colorimeter. As can be seen from Table 4, the values a* and b* for the Asconi samples are similar, with significant differences being recorded for the L values, and the A-FD sample having a higher brightness. The Cricova samples showed significant differences both for brightness (with the C-FD sample having a higher brightness) and for the a and b values. The C-FD sample has a higher brightness than the C-OD sample and a more pronounced reddish tint. Similar values were obtained for the Efes samples, where the freeze-dried sample registered a higher brightness than the dry one in the oven, but with lower values of a* and b*. The drying methods used in this study showed that the samples dried by freeze-drying have a higher luminosity value compared to the samples dried in the oven, registering similar values depending on the drying method used. Freeze-drying preserves the color of samples better due to low temperatures and no oxygen, protecting sensitive pigments such as anthocyanins and carotenoids. Oven-drying can significantly alter color due to high temperatures and exposure to oxygen, which favors pigment degradation, oxidation of phenolic compounds, and browning reactions, leading to discoloration or darkening of the color. A comparative study between hot-air-drying and freeze-drying on the anti-oxidant activity, color, and certain nutritional characteristics of strawberries, established that low temperature and a vacuum reduce the thermal degradation of heat-sensitive pigments (such as anthocyanins) and limit non-enzymatic browning or Maillard reactions—both known to cause discoloration during heat drying [50]. Similar results were identified for the L parameters in the lyophilized pumpkin samples compared to air-drying [51]. Also, the redness (a* values) is better preserved, helping maintain a color close to the fresh state (from 13.43 in FD to 11.12 in OD). By contrast, the redness in our samples increased from ~1 to 9 in the oven-dried samples due to Maillard-related changes or anthocyanin breakdown, which degraded the original color.

### 2.6. Mineral Content

There are many known uses of yeast biomass, including its potential for the bioremediation of heavy metals, regardless of whether the development environment is more or less favorable [52]. Yeast contamination with metals during the wine-making process comes from ripe grapes (primary contamination from the soil that is then passed into the grapes), from the administration of various treatments to the soil or fruit (secondary), or from the nature of the winemaking process (contact with containers and fining/clarification solutions). A detailed description of the origin and types of metals predominantly found in wine was provided by Pohl (2007) [53].

To ensure adequate nutrition in terms of mineral substances, and to eliminate the risks of contamination with heavy metals from yeast, a flame absorption spectrometric analysis was performed on the samples dried by the two methods described above (oven-drying and freeze-drying). Five types of metals were analyzed: zinc, sodium, calcium, iron, and copper. Their choice was made according to their prevalence in the winemaking process and implicitly in yeast: calcium and sodium are important in obtaining efficient fermentation, and come from the soil and from the clarification substances. On the other hand, copper, iron, and zinc are indicators of the use of pesticides/fungicides, and are intensively used by yeast in metallo-enzymatic processes [53,54].

The results of the atomic absorption spectrometry analyses are shown in Table 5.

As can be seen in Table 5, the experimental results are within the linearity range, above the detection and quantification limits established in the validation protocol, certifying the accuracy and precision of the laboratory results. The LOD/LOQ ratio has been verified, and the data validated. By comparing the data resulting from the analyses of Vieira et al. (2016) regarding the mineral content of spent brewer’s yeast, a reduced sodium content of up to 250 times (12.28 g/kg Na), calcium content of up to 5 times (271 mg/kg Ca), and zinc content of up to 6 times (119 mg/kg Zn), was observed in the analyzed samples. In contrast, iron concentrations are close to or slightly increased (from 14 to 47 mg/kg compared to the previously discovered 17 mg/kg Fe), and for copper, the data vary from 1.29 mg/kg to 64.71 mg/kg (3.64 mg/kg Cu) [30]. A significant deviation in copper concentration was observed in C-OD and C-FD samples, an indicator that the yeast accumulated copper because of the cultural practices applied in the winemaking process. Sancho-Galán et al. (2020) also analyzed the chemical composition of wine lees from three different types of wine (white, rosé, and red) for possible use in the food industry [55]. Their results identified similar values of calcium compared to the samples examined (between 18 and 105 mg/L), and copper ranged between 1.48 and 4.11 mg/L. In contrast, the authors identified lower values of sodium (3–4.41 mg/L) and iron (between 0.7 and 2.6 mg/L), while the highest concentration of zinc was at a value of 0.815 mg/L. Regarding the experimental results obtained, there are no significant differences between the freeze-dried and oven-dried samples (only in three cases were deviations of the values from 97 mg/kg in the oven-dried sample to 51 mg/kg Na in the freeze-dried sample, and also from 3 to 15 mg/kg Cu and from 21 mg/kg in the freeze-dried sample to 40 mg/kg Fe in the oven-dried sample). Also, close values were identified between the spent brewer’s yeast and wine lees samples.

In nutrition, various metals play vital roles, both as essential nutrients and as potential contaminants. Essential metals like copper, zinc, iron, manganese, and selenium are crucial for productivity (Cu and Fe prevent anemia, Zn provides appropriate feather development and eggshell deposition, Na is crucial for regulating water balance, and Ca has multiple uses in bone formation, egg formation, coagulation, or cell signaling), while others like cadmium, lead, mercury, and chromium can be harmful [56]. Habib et al. (2020) analyzed the influence of iron and copper by adding them to the drinking water of broiler chickens on immunity. The authors’ results, by administering Fe up to 200 ppm and Cu up to 50 ppm, revealed a significant increase in immunity but also the production of antibodies against the Newcastle virus [57]. To sum up briefly, these data suggest that the wine lees and spent brewer’s yeast analyzed provide adequate mineral content and do not exceed the levels at which a particular mineral may be toxic.

### 2.7. Determination of Organic Acid Content

Wine yeast (in particular, *Saccharomyces cerevisiae*) does not directly contain organic acids in the sense that it has them as a fixed part of the composition; however, wine lees and spent brewer’s yeast (dead yeast cells and other particles deposited after fermentation) contain organic acids from metabolic residues in yeast, from residual acids in wine or as a result of compounds released at yeast autolysis [58,59]. The combination of organic acids naturally present in wine and those produced or retained by yeast make wine lees a source of organic acids [60]. Both types of WL and SBY are interesting sources of organic acids, with potential for use in nutraceuticals, natural preservatives, and biotechnological applications. The results are similar for the two by-products, both for the freeze-drying method and for the oven-drying method, as can be seen in Table 6. Only for succinic acid has there been reported a difference between the two drying methods, but it is found in the highest quantity, being the main organic acid produced by yeast, accumulating and remaining partially in the inactive biomass [61].

### 2.8. Microstructure Analysis

**Figure 4:**
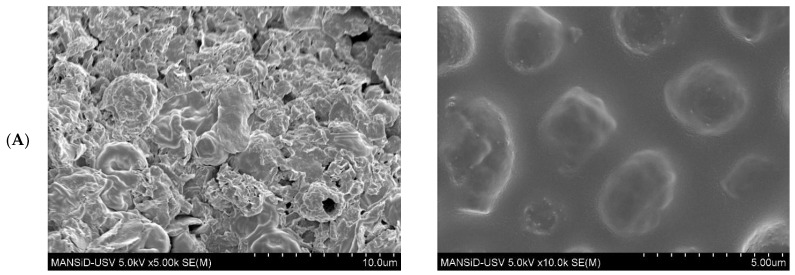

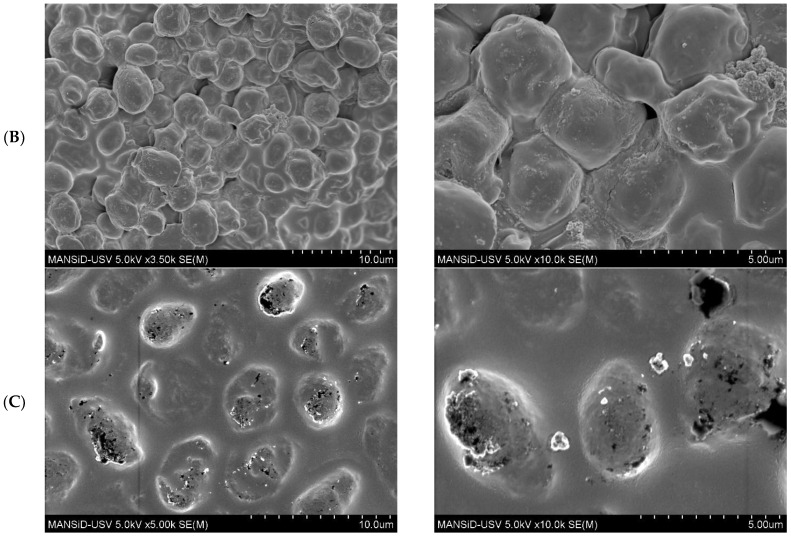
The surface morphology of freeze-dried yeast examined by FE-SEM at 3.5 k×, 5 k×, and 10 k×. (**A**) Asconi wine lees (A-FD); (**B**) Cricova wine lees (C-FD); and (**C**) Efes spent brewer’s yeast (E-FD).

In general, yeasts are ellipsoidal in shape with diameters ranging from 3 to 5 µm, and their morphology varies depending on the environmental condition in which they grow [62]. The Asconi sample is presented as autolyzed and wrinkled cells, indicating the release of intracellular contents (Figure 4).

Figure 4B,C display cells with a nearly perfect spherical shape that support better nutrient preservation. In Figure 4B, the spent yeast appears in the form of spores, suggesting that latent structures developed under limited nutrient conditions, since the well-defined morphology indicates enhanced resistance to environmental stress. At 10 k× magnification (on the right column), bridges connecting adjacent cells can be observed. Coluccio & Neiman (2004) suggested that these bridges may consist of chitin and dityrosine [63]. Distinct budding scars are also visible. The agglomerates and irregular structures in the Asconi yeast sample in Figure 4A may indicate autolysis induced during storage (similar to the morphological changes caused by various yeast treatments) [64]. Additionally the prominence of glucans in the cell wall after the freeze-drying process appears concentrated in layers with a porous surface [5,65].

## 3. Materials and Methods

### 3.1. Preparation of Spent Brewer’s Yeast and Wine Lees Flours

This study used two types of wine lees from Asconi Winery, Republic of Moldova (19% DW) and Cricova Winery, Republic of Moldova (23% DW). The spent brewer’s yeast was kindly offered by Efes Vitanta Moldova Brewery, Republic of Moldova (16% DW). The samples of SBY and WL were dried in the oven or subjected to freeze-drying, until the weight change was <0.1% over a 1 h period. Yeast samples were dried by oven-drying at 55 °C using a forced-air-drying Zhicheng ZRD-A5055 oven (Zhicheng, Shanghai, China), while the freeze-dried process of the samples was performed under −40 °C and a pressure of <10 Pa (Biobase BK-FD12S Freeze-Dryer, Jinan, China) for 24 h up to 98% dry weight. The samples were labeled as follows: Asconi freeze-dried yeast (A-FD), Asconi oven-dried yeast (A-OD), and so on (Cricova: C-FD, C-OD; Efes: E-FD, E-OD). All chemicals used in this paper were of analytical grade and were purchased from Merck Group (Darmstadt, Germania).

### 3.2. Proximate Composition

Moisture content was determined by drying the samples at 105 °C until a constant weight was achieved in an oven (Memmert, 391 GmbH&Co. KG, Schwabach, Germany). The moisture content was defined as described below:

$$\mathrm{Moisture}\;=\;\frac{m_{0}-m}{m_{0}}\times 100$$

where m_0_ = initial weight (g) and m = final dry weight (g).

Ash content was determined in a calcination furnace at 550 °C for 12 h. The Kjeldhal method was used to estimate protein content, while the Soxhlet method was used to determine lipid content with petroleum ether solvent. Using a digital pH meter (Mettler-Toledo GmbH, Greifensee, Switzerland), the SBY and WL samples were subjected to direct pH readings.

### 3.3. FT-IR Analysis

A Nicolet iS10 spectrometer from Thermo Scientific (Karlsruhe, Dieselstraòe, Germany) fitted with a ZnSe crystal and an attenuated total reflectance (ATR) accessory was utilized for Fourier Transform Infrared Spectroscopy (FTIR) investigation. In the mid-infrared range of 650–4000 cm^−1^, measurements were made using the reflecting absorbance mode (ATR-FTIR) with 32 scans in transmission mode and a resolution of 4 cm^−1^. Dried samples of SBY and WL were placed on the ATR crystal and the OMNIC software (version 32, Thermo Fisher Scientific Inc., Waltham, MA, USA) was used for spectra acquisition.

### 3.4. Total Phenolic Content (TPC)

The Folin-Ciocalteau method was used to determine the phenolic content, as explained: in a tube, 0.2 mL of extract (made of 3 g SBY/WL:30 mL ethanol, sonicated and centrifuged to obtain the supernatant), 2 mL of a 1:10 diluted Folin-Ciocalteau reagent, and 1.8 mL of 7.5% (*w*/*v*) sodium carbonate were added. The combination was allowed to sit at room temperature in the dark for half an hour. Total phenolic compounds were measured at a wavelength of 750 nm, using a Shimadzu 3600 UV-VIS-NIR spectrophotometer (Shimadzu Corporation, Kyoto, Japan). Gallic acid was used to measure the calibration curve of the polyphenols at concentrations ranging from 10 to 200 mg/L, with a regression coefficient R^2^ = 0.9987.

### 3.5. 2,2-Diphenyl-1-Picrylhydrazyl (DPPH) Assay Scavenging Activity

In order to assess the 2,2-diphenyl-1-picrylhydrazyl (DPPH) scavenging activity, 2 mL of each extract was mixed with 2 mL of DPPH solution 0.1 mM in methanol. A UV-VIS-NIR spectrophotometer (Shimadzu Corporation Kyoto, Japan) was used to measure the absorbance at 517 nm after the mixture had been shaken for two minutes and allowed to sit at room temperature in a dark area for thirty minutes.

% inhibition of DPPH = [(1 − As/Ab)] × 100

where As = absorbance of the sample and Ab = absorbance of the blank sample.

### 3.6. Analysis of Individual Phenolic Compound by HPLC-DAD

A High-Performance Liquid Chromatography (HPLC) system (Shimadzu, Kyoto, Japan) with an SPD-M-20A diode array detector, SIL-20A auto sampler, CTO-20AC column oven, and LC-20 AD liquid chromatograph was used for the analysis. A Phenomenex Kinetex^®^ 2.6 μm Biphenyl 100 Å HPLC Column 150 × 4.6 mm, thermostated at 25 °C, was used for the separation. The injection volume of the extracts was 10 µL. A solvent system comprising 0.1% acetic acid in water (solvent A) and acetonitrile (solvent B) was employed with the following gradient: starting with 100% A, a gradient was installed to obtain 5% B at 6.66 min, 40% B at 66.6 min, and 80% B at 74 min. One milliliter per minute was the solvent flow rate. Extracts were filtered via 0.45 µm pores before injection. By comparing the absorbance of the chromatograms to external standards, which were 280 nm for gallic acid, protocatechuic acid, vanillic acid, and p-hydroxibenzoic acid, and 320 nm for chlorogenic acid, caffeic acid, p-coumaric acid, rosmarinic acid, myricetin, quercetin, luteolin, and kaempherol, phenolic compounds were identified based on the retention times of standard materials. High levels of linearity were displayed by all standard calibration curves (R^2^ > 0.99).

### 3.7. Color

CIELab color space coordinates, where L* values represent black to white (0 to 100), a* is the degree of redness (positive) or greenness (negative), and b* is yellowness (positive) or blueness (negative), were used to quantify the color using a Konica Minolta CR-400 colorimeter (Tokyo, Japan).

### 3.8. Mineral Content

The mineral content of SBY and WL samples was determined by using atomic absorption spectroscopy (AAS, Shimadzu AA-6300, Japan). High-purity reagents (HNO_3_, Merck, Germany, Suprapure grade) were used to dissolve the ashes obtained after calcination at 550 °C (heating above this temperature will significantly reduce the element recovery). For the analysis (AAS), a multi-element tube cathode lamp and air flame/acetylene were employed. The concentration of calcium (Ca), iron (Fe), zinc (Zn), copper (Cu), and sodium (Na) were quantified. Each determination was performed in triplicate for accuracy. Stock standard solutions (1000 mg/L) of Ca (Fluka 38995), Fe (Fluka 16596), Zn (Fluka 18827), Cu (Fluka 38996), and Na (Fluka 05201) were used for calibration. Calibration curves were constructed using five concentration points: 0.05–1 mg/L for Na, Cu, and Fe; 0.5–5 mg/L for Ca; and 0.05–0.6 mg/L for Zn. Mineral concentrations in the samples were calculated directly, considering the volume, weight, and dilution factors.

### 3.9. Determination of Complex B Vitamins Content

The High-Performance Liquid Chromatography (HPLC) system consisted of a SCL-40 system controller (Shimadzu, Kyoto, Japan), a LC-40B X3 solvent delivery module (Shimadzu), a SIL-40C X3 auto-sampler (Shimadzu), a CTO-40C column oven (Shimadzu), and an SPD-M40 photo diode array detector (Shimadzu). For vitamin B analysis, a Phenomenex Kinetex^®^ 5 μm C18 100 Å HPLC Column 250 × 4.6 mm, conditioned at 30 °C, was used for the separation. The injection volume of the sample was 10 µL. A solvent system comprising 0.0125 M hexane-1-sulfonic acid sodium salt in 0.1% (*m*/*v*) o-phosphoric acid, pH adjusted at 2.5 (solvent A) and acetonitrile (solvent B), was employed with the following gradient: starting with 100% A for 15 min, changing to 85% A and 15% B for the next 20 min, being held constant for 5 min, then being set to 70% A and 30% B over the next 15 min, being held constant for 5 min, and finally being set to 100% A for 1 min, as described by [38]. Standard curves for vitamins were made at five points over a range of 0.06–1.2 mg/mL [40] (0.06, 0.19, 0.33, 0.50, and 1.2 mg/mL), made from a mix of five vitamins by dissolving an accurate amount of reference standard in MilliQ water (B1, B2, B3, B6, and B12), while B7 was quantified separately due to the use of a pH 9 buffer in the preparation of the stock solution [39].

Sample preparation for analysis was carried out according to Mateeva et al. (2023), where 1 g of yeast was treated with 26 mL 1 N NaOH, incubated for one hour at 50 °C, acidified to pH 5.6, and centrifuged at 4000 rpm; finally, the supernatant obtained was filtered twice for injection into HPLC [39].

### 3.10. Determination of Organic Acid Content

The organic acids in SBY and WL were determined using the following method adapted by Pauliuc & Oroian (2020): 0.5 g of sample is mixed with 2.5 mL 4% metaphosphoric acid (*w*/*v*), stirred and centrifuged for 5 min at 3500 rpm, and filtered through 0.45 µm syringe filters [66]. The mobile phase, under continuous gradient elution, consisted of a mixture of 0.5% metaphosphoric acid and acetonitrile at a flow rate of 0.8 mL/min on a Phenomex Kinetex column, 5 µm C18 A 250 × 4.6 mm, with HPLC equipment (Shimadzu, Kyoto, Japan) equipped with photodiode string detector SPD-M-20A.

### 3.11. Microstructure

The morphology of the SBY and WL was investigated by means of Field Emission Scanning Electron Microscopy (FE-SEM) (SU-70, Hitachi, Tokyo, Japan), operating at an accelerating voltage of 5 kV. The yeast samples were deposited on a thin layer of carbon double-sided adhesive tapes. Before analysis, the samples were covered with a conductive gold film of about 2 nm and then examined.

### 3.12. Fiber Content

To evaluate the fiber content, a FibroTherm FT12 device (Gerhardt GmbH & Co. KG, Königswinter, Germany) was used in combination with a Megazyme K-TDFR-200A kit (Megazyme Ltd., County Wicklow, Ireland) following the AOAC 985.29 method [67].

The total fiber content was determined using the following formula:

$$Fiber=\frac{m_{2}-m_{3}-m_{4}}{m_{1}}\times 100$$

where

m_1_ = mass of the initial sample;
m_2_ = mass of the bag with the residue remaining after digestion;
m_3_ = mass of the empty bag;
m_4_ = mass of the bag with the residue remaining after calcination.

### 3.13. Statistical Analysis

In the current study, all measurements were performed in triplicate. Results are expressed as means ± standard deviation (SD). Statistical analysis of the data was conducted using XLSTAT for Excel 2021 (Addinsoft, New York, NY, USA). One-way ANOVA, followed by Tukey’s test, was performed to assess significant differences (*p <* 0.05) between samples.

## 4. Conclusions

The study evaluates the nutritional and functional potential of spent brewer’s yeast (SBY) and wine lees (WL). Notably, the highest protein concentrations were found in wine lees, indicating its potential as an alternative protein source. Polyphenolic compounds were mainly abundant in brewer’s yeast and freeze-dried samples, while antioxidant activity was not strictly linked to total polyphenol content, implying that non-phenolic compounds also play a role. Five of the six B vitamins were quantified effectively, with freeze-drying aiding their preservation.

Colorimetric and microstructural analyses showed that freeze-dried samples had better luminosity and preserved cellular structures compared to other methods, which exhibited signs of autolysis. Mineral analysis confirmed adequate levels of essential minerals such as calcium, sodium, iron, zinc, and copper for nutritional applications, remaining below toxic thresholds. The identification of organic acids like succinic, acetic, gluconic, and formic acids suggests these by-products could be valuable for nutraceutical and technological uses.

Overall, freeze-drying outperformed oven-drying in preserving vitamins and cell structure, although both methods yielded comparable nutritional profiles. The findings underscore SBY and WL as promising sources of proteins, polyphenols, vitamins, and minerals for biotechnology and food industry applications. However, further studies are necessary to elucidate their composition and optimize valorization strategies, as the results vary based on processing technology and input materials. Variations in raw materials, fermentation conditions, microbial communities, and processing techniques across different production sites could influence the chemical profiles observed. Therefore, studies based on a broader and more diverse range of producers would be necessary to confirm whether the trends identified here are consistent across the industry. The limitation of purine quantification is noted as a future research avenue.

## Figures and Tables

**Table 1:** Physicochemical parameters, TPC, and DPPH values of analyzed samples.

	A-FD	A-OD	C-FD	C-OD	E-FD	E-OD
Ash (%)	5.53 ± 0.21 ^c^	5.09 ± 0.17 ^b^	4.02 ± 0.09 ^a^	3.86 ± 0.1 ^a^	6.69 ± 0.11 ^d^	5.64 ± 0.08 ^c^
Lipid (%)	0.16 ± 0.03 ^a^	0.32 ± 0.01 ^b^	0.31 ± 0.01 ^b^	0.32 ± 0.01 ^b^	0.17 ± 0.01 ^a^	0.28 ± 0.01 ^b^
Protein (%)	13.48 ± 0.02 ^b^	12.86 ± 0.03 ^a^	46.81 ± 0.19 ^f^	45.35 ± 0.21 ^e^	41.58 ± 0.25 ^d^	29.28 ± 0.18 ^c^
TPC (mg GAE/g DW)	1.57 ± 0.04 ^a^	1.86 ± 0.02 ^b^	2.4 ± 0.02 ^c^	3.05 ± 0.03 ^d^	3.15 ± 0.02 ^e^	3.98 ± 0.01 ^f^
DPPH (% inhibition)	88.92 ± 0.23 ^e^	88.42 ± 0.16 ^e^	77.34 ± 0.14 ^d^	46.31 ± 0.20 ^c^	33.74 ± 0.19 ^a^	44.33 ± 0.23 ^b^
Fiber content (%)	10.66 ± 0.02 ^f^	7.27 ± 0.03 ^e^	0.19 ± 0.05 ^a^	0.43 ± 0.05 ^b^	6.88 ± 0.03 ^d^	2.67 ± 0.07 ^c^

E-OD: Efes oven-dried yeast; E-FD: Efes freeze-dried yeast; A-FD: Asconi freeze-dried yeast; A-OD: Asconi oven-dried yeast; C-OD: Cricova oven-dried yeast; and C-FD: Cricova freeze-dried yeast. Mean values with different letters in the same row are significantly different (*p* < 0.05).

**Table 2:** Content of individual polyphenols on dry mass basis determined in wine lees and brewer’s spent yeast.

Individual Phenolics (mg/kg)	A-FD	A-OD	C-FD	C-OD	E-FD	E-OD
4- hidroxibenzoic acid	20.58 ± 0.03 ^b^	8.79 ± 0.02 ^a^	142.46 ± 0.15 ^f^	64.40 ± 0.04 ^c^	105.17 ± 0.13 ^e^	97.33 ± 0.08 ^d^
Vanillic acid	N.D.	7.30 ± 0.01 ^c^	28.84 ± 0.09 ^e^	23.50 ± 0.01 ^d^	0.98 ± 0.01 ^b^	123.8 ± 0.18 ^f^
Caffeic acid	N.D.	N.D.	16.29 ± 0.05 ^d^	4.78 ± 0.01 ^d^	3.04 ± 0.01 ^b^	26.55 ± 0.11 ^e^
Chlorogenic acid	N.D.	N.D.	N.D.	N.D.	N.D.	4.27 ± 0.06 ^b^
p-coumaric acid	N.D.	N.D.	0.52 ± 0.01 ^b^	N.D.	0.90 ± 0.01 ^c^	3.13 ± 0.01 ^d^
Rosmarinic acid	62.25 ± 0.04 ^c^	3.84 ± 0.02 ^b^	82.21 ± 0.16 ^f^	22.13 ± 0.07 ^d^	4.45 ± 0.02 ^c^	N.D.
Miricetin	N.D.	N.D.	N.D.	N.D.	0.96 ± 0.02 ^b^	N.D.
Luteolin	N.D.	N.D.	N.D.	N.D.	1.52 ± 0.04 ^b^	N.D.
Quercitin	0.92 ± 0.01 ^b^	N.D.	N.D.	N.D.	N.D.	N.D.
Kaempferol	3.48 ± 0.02 ^b^	12.85 ± 0.04 ^c^	N.D.	N.D.	N.D.	N.D.

Mean values with different letters in the same row are significantly different (*p* < 0.05); N.D.—Not detected.

**Table 3:** Vitamin content of the wine lees and spent brewer’s yeast samples (*n* = 3).

Sample	Vitamers (mg/g DW)					
	B1 (Thiamine Hydrochloride)	B2 (Riboflavin)	B3 (Nicotinamide)	B6 (Pyridoxine Hydrochloride)	B7 (Biotin)	B12 (Cyanocobalamin)
A-OD	0.0404 ± 0.025 ^ab^	0.0795 ± 0.043 ^a^	2.0265 ± 0.016 ^e^	0.0888 ± 0.008 ^abc^	0.0237 ± 0.003 ^b^	N.D.
A-FD	0.0711 ± 0.007 ^bc^	0.0196 ± 0.003 ^a^	2.3845 ± 0.070 ^f^	0.1176 ± 0.048 ^bc^	0.0100 ± 0.001 ^a^	N.D.
C-OD	0.0274 ± 0.018 ^a^	0.0357 ± 0.003 ^a^	0.8667 ± 0.038 ^a^	0.0499 ± 0.023 ^a^	0.0096 ± 0.000 ^a^	N.D.
C-FD	0.0400 ± 0.007 ^ab^	0.0362 ± 0.005 ^a^	1.1343 ± 0.022 ^b^	0.0524 ± 0.022 ^ab^	0.0092 ± 0.001 ^a^	N.D.
E-OD	0.0676 ± 0.005 ^bc^	0.0730 ± 0.046 ^a^	1.3559 ± 0.005 ^c^	0.1230 ± 0.012 ^c^	0.0296 ± 0.001 ^c^	N.D.
E-FD	0.0904 ± 0.006 ^c^	0.0486 ± 0.005 ^a^	1.5480 ± 0.025 ^d^	0.0998 ± 0.000 ^abc^	0.0095 ± 0.000 ^a^	N.D.

Mean values with different letters in the same column are significantly different (*p* < 0.05); N.D.—Not detected.

**Table 4:** Wine lees and spent brewer’s yeast sample color parameters.

Sample	L	a*	b*
A-OD	35.77 ± 0.04 ^a^	1.69 ± 0.01 ^b^	9.25 ± 0.02 ^a^
A-FD	52.14 ± 0.18 ^d^	1.14 ± 0.01 ^a^	10.05 ± 0.04 ^b^
C-OD	42.66 ± 0.24 ^c^	9.41 ± 0.03 ^f^	24.18 ± 0.06 ^e^
C-FD	53.99 ± 0.09 ^e^	2.04 ± 0.01 ^c^	14.32 ± 0.08 ^c^
E-OD	39.55 ± 0.11 ^b^	9.28 ± 0.04 ^e^	24.30 ± 0.02 ^e^
E-FD	63.59 ± 0.21 ^f^	2.33 ± 0.02 ^d^	16.45 ± 0.01 ^d^

Mean values with different letters in the same column are significantly different (*p* < 0.05).

**Table 5:** Concentration of chemical elements (mg/kg, dry weight basis) in yeast samples with reported values using F-AAS.

	Sample								
Elements	A-FD (mg/kg)	A-OD (mg/kg)	C-FD (mg/kg)	C-OD (mg/kg)	E-FD (mg/kg)	E-OD (mg/kg)	LOD (mg/L)	LOQ (mg/L)	R^2^
Zn	17.2726	17.9297	32.1268	29.6922	26.9249	36.6846	0.0424	0.1286	0.9963
Na	88.5243	77.4087	66.4684	61.1788	51.6377	97.0966	0.0997	0.3022	0.9897
Ca	144.8609	115.1698	61.1422	62.1131	52.9292	78.1660	1.3568	4.1116	0.9366
Fe	21.4996	40.0418	14.4199	25.0583	40.3651	47.5014	0.0758	0.2297	0.9940
Cu	2.2919	1.2932	64.5609	64.7108	15.7095	3.1455	0.0865	0.2620	0.9922

E-OD: Efes oven-dried yeast; E-FD: Efes freeze-dried yeast; A-FD: Asconi freeze-dried yeast; A-OD: Asconi oven-dried yeast; C-OD: Cricova oven-dried yeast; C-FD: Cricova freeze-dried yeast; LOD: Limit of Detection; LOQ: Limit of Quantitation; and R^2^: coefficient of determination.

**Table 6:** Organic acid content in wine lees and brewer’s spent yeast.

Organic Acid (g/kg DW)	A-FD	A-OD	C-FD	C-OD	E-FD	E-OD
Gluconic acid	0.31 ± 0.05 ^b^	0.32 ± 0.03 ^b^	1.09 ± 0.02 ^d^	1.38 ± 0.02 ^e^	0.81 ± 0.05 ^c^	0.11 ± 0.01 ^a^
Formic acid	0.12 ± 0.07 ^a^	0.14 ±0.01 ^a^	0.26 ± 0.03 ^b^	0.73 ± 0.04 ^d^	0.41 ± 0.02 ^c^	0.17 ± 0.01 ^ab^
Acetic acid	0.17 ± 0.06 ^a^	0.21 ± 0.07 ^ab^	0.68 ± 0.03 ^d^	0.42 ± 0.03 ^c^	0.31 ± 0.01 ^bc^	0.24 ± 0.04 ^ab^
Succinic acid	N.D.	N.D.	1.11 ± 0.01 ^c^	2.56 ± 0.09 ^d^	0.19 ± 0.01 ^b^	1.02 ± 0.01 ^c^
Lactic acid	N.D.	N.D.	N.D.	N.D.	N.D.	N.D.
Total organic acids	0.6	0.67	3.14	5.09	1.72	1.54

Mean values with different letters in the same row are significantly different (*p* < 0.05); N.D.—Not detected.

## Data Availability

The original contributions presented in this study are included in the article. Further inquiries can be directed to the corresponding author.
